# Near-field terahertz nonlinear optics with blue light

**DOI:** 10.1038/s41377-023-01137-y

**Published:** 2023-04-19

**Authors:** Angela Pizzuto, Pingchuan Ma, Daniel M. Mittleman

**Affiliations:** 1 Department of Physics, Brown University, Providence, RI 02912 USA; 2 School of Engineering, Brown University, Providence, RI 02912 USA

**Keywords:** Terahertz optics, Sub-wavelength optics, Nonlinear optics

## Abstract

The coupling of terahertz optical techniques to scattering-type scanning near-field microscopy (s-SNOM) has recently emerged as a valuable new paradigm for probing the properties of semiconductors and other materials on the nanoscale. Researchers have demonstrated a family of related techniques, including terahertz nanoscopy (elastic scattering, based on linear optics), time-resolved methods, and nanoscale terahertz emission spectroscopy. However, as with nearly all examples of s-SNOM since the technique’s inception in the mid-1990s, the wavelength of the optical source coupled to the near-field tip is long, usually at energies of 2.5 eV or less. Challenges in coupling of shorter wavelengths (i.e., blue light) to the nanotip has greatly inhibited the study of nanoscale phenomena in wide bandgap materials such as Si and GaN. Here, we describe the first experimental demonstration of s-SNOM using blue light. With femtosecond pulses at 410 nm, we generate terahertz pulses directly from bulk silicon, spatially resolved with nanoscale resolution, and show that these signals provide spectroscopic information that cannot be obtained using near-infrared excitation. We develop a new theoretical framework to account for this nonlinear interaction, which enables accurate extraction of material parameters. This work establishes a new realm of possibilities for the study of technologically relevant wide-bandgap materials using s-SNOM methods.

## Introduction

The advent of scattering-type scanning near-field optical microscopy (s-SNOM) in the mid-1990s has revolutionized the field of subwavelength optics^1,2^. This technique involves the coupling of electromagnetic radiation to a sharp subwavelength metal tip, held near a surface, and the subsequent measurement of the radiation scattered from this tip-sample junction in the far field. Over the last decade, this approach to near-field measurement has had a notable impact in the infrared and terahertz regions of the spectrum, where aperture-based methods for subwavelength spectroscopy are challenging^3^. Coupling the incident wave to the metal tip becomes easier as the wavelength increases, while the spatial resolution remains tip-size-limited^4,5^. These s-SNOM experiments are also compatible with time-domain measurements^6,7^, which has opened up exciting new possibilities for time- and spatially resolved spectroscopy^8,9^, as well as other nonlinear optical studies^10–12^. In general, though, these efforts remain limited to longer-wavelength regimes, as coupling of short-wavelength radiation to the nanoscale tip remains a daunting task; this has hindered the nanoscale study of important wide-bandgap materials such as Si and GaN^13–15^. Such materials have been investigated in the near-field using below-band-gap excitation, in linear optical studies^16–18^. Yet, the nanoscale nonlinear optical methods that have become so prominent in their application to other materials^19,20^ have so far not been applied to these highly relevant material systems, since these methods would in general require higher energy photoexcitation.

Here, we describe the first s-SNOM measurement in which the incident photon energy exceeds 3 eV. Using femtosecond pulses at 410 nm, we illuminate a sharp metallic atomic force microscope (AFM) tip and induce terahertz emission from several different materials via a second-order nonlinear optical process, to perform laser terahertz emission microscopy (LTEM) with nanoscale spatial resolution^11^. The high energy of the pump photons enables strong THz emission from bulk crystalline silicon due to two-photon excitation above the wide direct bandgap. The unique aspect of terahertz LTEM leads to a considerably relaxed requirement for optical alignment; traditional linear s-SNOM uses the nano-tip to confine the incident wave, and this precise alignment of focused short wavelength radiation under the nano-tip is practically challenging. In this experiment, however, nanoscale resolution is obtained through out-coupling of a small fraction of the much larger *macroscopic* photo-generated terahertz dipole, thus enabling the use of tightly focused blue light in s-SNOM for the first time. We perform the first near-field LTEM image of Si and compare the results to those obtained with THz s-SNOM via elastic scattering of THz pulses from the tip^21^. Our results open new possibilities for the application of s-SNOM methods to wide-bandgap materials.

## Results

### THz emission from InAs

**Fig. 1:**
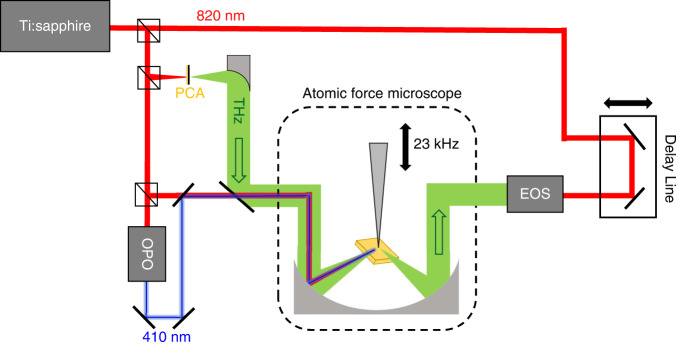
Schematic of laser path and s-SNOM experiment. NIR, blue light, and THz beams are generated separately, with the THz pulses generated using a conventional photoconductive antenna (PCA). All three beams then overlap and are coupled into the AFM. Scattered or emitted THz pulses are coherently detected on the other side via free-space electro-optic sampling (EOS)

**Fig. 2:**
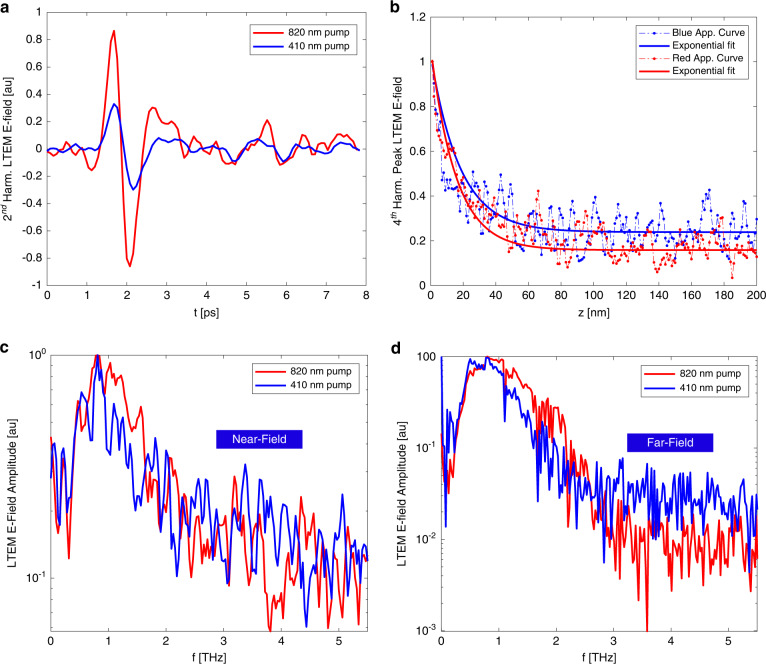
Near and far field THz emission induced via NIR and blue light from p-InAs. **a** Time-domain THz emission waveforms from an NIR pump (red) and blue light pump (blue), measured at 2nd harmonic demodulation. **b** Associated 4th harmonic approach curves showing strong field confinement. **c** Near-field THz emission spectra from p-InAs, from the waveforms shown in (**a**). **d** Far-field THz emission spectra from p-InAs, referenced to an optical chopper which chops the laser beam at ~3 kHz

We begin our study of LTEM via blue light using a wafer of lightly *p*-doped bulk InAs. Its bandgap is direct and very narrow^22^ (~0.35 eV) making photoexcitation easy in both the NIR and visible regimes. InAs has been shown to be a strong THz emitter in both the far-field^23^ and the near-field^10,11^ via the Photo-Dember effect, in which photoexcitation generates a vertical subsurface dipole and produces picosecond THz pulses (Fig. 1). As shown in Fig. 2a, we observe strong THz emission in the near field induced by both NIR and blue pump pulses. We measure approach curves (shown at 4th harmonic demodulation in Fig. 2b) in which the peak emission signal is measured as a function of the distance between the tip and sample surface. We observe exponential-type decay in both cases, which is characteristic of strong near-field confinement^24,25^. The decay widths of these curves can be used to estimate the size of the confined radiation near the tip apex, and consequently the associated imaging resolution. We perform exponential fits of both curves and find 1/*e* widths of 16.6 ± 1.1 nm for NIR-pumped emission and 13.2 ± 1.8 nm for blue-pumped emission (with a somewhat higher noise floor due to the smaller signal strength as seen in Fig. 2a). We note that this 1/*e* width is smaller than the specified tip radius of ~40 nm; this is consistent with previous studies which have shown^10,26^ that the tip radius is not a perfect metric for determining the true field confinement or the highest achievable spatial resolution; the true relationship between these quantities is more complex, and in this case, the nonlinearity of the LTEM experiment further confounds this relationship. However, the 1/*e* width is an excellent method for tracking *trends* in the spatial resolution as a function of harmonic demodulation order, and so these results strongly suggest that the observed signals originate from the near field of the tip, with adequate background suppression and tip-limited field confinement. The waveforms in Fig. 2a suggest that the THz pulse induced via blue light is slightly broader than in the NIR-pump case, and this is confirmed by the spectra in Fig. 2c; the blue-light-induced THz spectrum is significantly narrower, suppressed at frequencies above 1 THz relative to the NIR-induced THz pulse. We also perform the same THz emission measurements in a far-field configuration, by referencing the lock-in amplifier to an optical chopper which modulates both the NIR and blue beams at 3.17 kHz (rather than to the tapping frequency of the AFM tip). The resulting far-field spectra, shown in Fig. 2d, show the same spectral narrowing. This unambiguously demonstrates that this change in the spectrum of the emitted THz signal is not induced by the geometry of the tip or the coupling from the near field to the far field, but rather is intrinsic to the THz generation mechanism in InAs. This result is not surprising; the higher 3.02 eV photon energy in the blue-light pump pulse excites electrons higher in the band, where they can rapidly scatter from the Γ to the L valley. In this satellite valley, electrons have an ~6 times higher effective mass^22^, resulting in lower mobility and less efficient THz emission. In contrast, NIR excitation at 1.51 eV, while still well above the bottom of the conduction band, is below the bottom of the L valley (*E* ≈ 1.86 eV) in InAs^22^, therefore inhibiting rapid Γ-L scattering^27^. This more rapid scattering in the case of blue-light pumping can explain both the lower emission efficiency and the narrower THz spectrum for 410 nm pump pulses compared to 820 nm pump pulses. We conclude that pumping with blue light can provide new information even in the case of a narrow-bandgap material, as the resulting emission may provide a more complete picture of the material’s charge carrier dynamics.

### THz emission from Si

To illustrate the value of using LTEM in a wide bandgap material, we switch from InAs to bulk single-crystal silicon, which does not emit significant THz radiation upon NIR excitation^28^. Our sample is a Si wafer which has a small area that has been subjected to ion implantation. Subsequent annealing has activated the implanted dopants in this area, such that the wafer contains two regions of very different doping densities, with a relatively sharp boundary between them. The unimplanted region is simply the unmodified Si substrate, which is lightly *n*-doped (concentration ~10^16 ^cm^−3^), while the boron-implanted region is *p*-doped, with a higher nominal doping density of about 10^18 ^cm^−3^. We perform both linear (THz imaging based on elastic scattering) and nonlinear (blue-light-induced THz emission) measurements of this boundary region and compare the results.

**Fig. 3:**
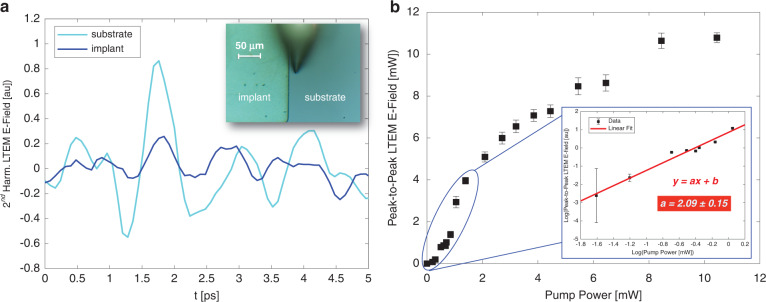
Terahertz emission from Si sample. **a** Near-field THz emission waveforms from a blue light pump, emitted by the Si substrate (light blue) and implanted region (dark blue). Inset: top-down AFM camera view of the implant-substrate boundary region. **b** Peak-to-peak far-field THz emission amplitude from the Si substrate region vs. 410 nm pump average power. Inset: log-log plot of low powers. Linear fit shows quadratic *E*-field vs. pump power behavior, suggesting the dominant mechanism of THz emission is two-photon absorption

First, we determine that both the unimplanted substrate and implanted area emit THz pulses when pumped with pulsed blue light (and, unsurprisingly, do *not* emit any measurable THz radiation when pumped with NIR pulses). The blue-light-induced THz pulses, demodulated at the 2nd harmonic of the tip tapping frequency, are shown in Fig. 3a. We observe that the lightly n-doped substrate produces significantly more THz emission than the more heavily p-doped implanted region. To better understand the THz generation mechanism, we measure the relationship between the emitted THz peak-to-peak amplitude and the average power of the blue pump beam (Fig. 3b). This measurement is done in the far-field, by referencing our lock-in amplifier to an optical chopper which chops the pump beam at 3.17 kHz. It is worth noting that the presence of the tip may affect the dielectric properties of the Si, but not appreciably enough to change the underlying mechanism of THz generation. Therefore, a far-field measurement is both convenient for an improved signal-to-noise ratio and appropriate to accurately represent the near-field photoexcitation process. We observe that when the pump power exceeds ~2 mW, the THz emission strength becomes less affected by an increase in blue light power; indeed, once the pump fluence is high enough, a large portion of available charge carriers will become photoexcited, and any excess pump photons will be screened by the high local conductivity. This saturation behavior has been observed in prior LTEM studies^11^. At low powers, however, the saturation effect is not dominant, and we see a clear quadratic relationship between the amplitude of the emitted THz field and the pump power (inset, Fig. 3b). This suggests that the primary mechanism of THz generation is two photon absorption; carriers in the valence band absorb over 6 eV of pump energy, and are excited high above the wide, 4.2 eV direct bandgap^28^ of bulk Si.

**Fig. 4:**
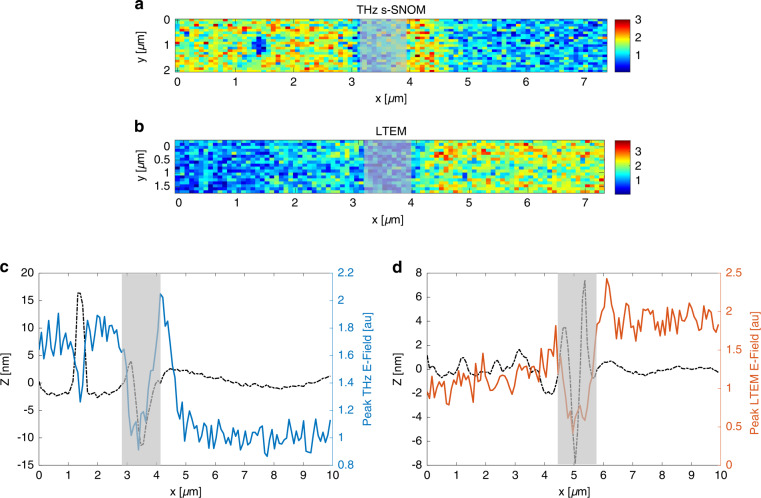
THz s-SNOM and blue light LTEM near-field images across implant boundary. **a** 2D THz s-SNOM image across the substrate/implant boundary, modulated at the 3rd harmonic of the tapping frequency. **b** LTEM image across the same boundary. **c** Average line profile from THz s-SNOM image in (**a**). **d** Average line profile from LTEM image in (**b**). Gray shaded regions mark boundary region where there is significant topography

We then fix the delay stage at the temporal peak of the emitted waveform, and raster-scan over the boundary between the substrate and the implant to produce a 2D THz emission image (10 µm x 2 µm area) (Fig. 4b). We then block the blue light and image the same area using an incident THz pulse (i.e., linear THz s-SNOM), measuring the peak THz reflection over the same region (Fig. 4a). We see contrasting behavior between the two images; the implanted region (left part of the images in Fig. 4a, b, and left part of the plots in Fig. 4c, d) reflects significantly more THz radiation than the more lightly doped substrate but emits significantly less. Averaged line profiles of the THz s-SNOM and emission images further illustrate this effect and are shown in Fig. 4c, d, respectively. In Fig. 4, the shaded regions represent the ~1 μm-wide boundary area between the unimplanted substrate and the implanted region. Because of the significant topographic features in this region (height profiles shown in Fig. 4c, d by the black dotted lines) the signals originating from inside this boundary region are difficult to interpret; it has been shown^26,29^ that significant topography over a short lateral range can introduce artifacts in THz near-field images. A small (~400 nm diameter) piece of debris is also visible in the THz s-SNOM image towards the left side (Fig. 4a, c), which introduces similar artifacts, and therefore we do not consider these areas in our subsequent analyses. The strong contrast in signal when comparing the flat regions on either side of the boundary is certainly representative of the difference in doping between the two regions. Moreover, although the trends are opposing, the magnitude of the contrast between the differently doped areas is very similar in Fig. 4c, d; that is, the implanted region produces ~1.7 times more THz reflection than the substrate, but about 1.9 times less THz emission. This suggests that near-field THz emission imaging of bulk Si with blue light may be used as an informative companion to the more well-established THz s-SNOM technique.

### Modifying the finite dipole model

To extract quantitative information from these measurements, a physical model of the near-field interaction is required. Several such models exist for accurately describing the linear elastic scattering; however, a framework which incorporates the in-sample THz generation process is still lacking. Here, we develop a new model to describe this nonlinear interaction, using the finite dipole model (FDM)^30^ as a starting point. This model approximates the AFM tip as a prolate spheroid held some height *H* above the sample surface. It then imposes an electrostatic dipole in the tip, induced by the incident THz field, and an image dipole which, by the method of images, contains the information about the dielectric function *ε* of the sample. The scattered near-field signal can be represented by the total far-field radiation of these two dipoles, as:^31^

1 $$\normalsize E_{THz\,s - SNOM} = \left( {1 + \beta } \right)\alpha _{eff}E_{inc}$$

where *E*_*inc*_ is the incident electric field, $\beta = \frac{{\varepsilon - 1}}{{\varepsilon + 1}}$, and *α*_*eff*_ is the effective polarizability of the tip and its image. The expression for parameter *α*_*eff*_ is:

2 $$\alpha _{eff} = R^2L\frac{{\frac{{2L}}{R} + \ln \frac{R}{{4eL}}}}{{\ln \frac{{4L}}{{e^2}}}}\left( {2 + \frac{{\beta \left( {g - \frac{{R + H}}{L}} \right)\ln \left( {\frac{{4L}}{{4H + 3R}}} \right)}}{{\ln \left( {\frac{{4L}}{{e^2}}} \right) - \beta \left( {g - \frac{{3R + 4H}}{{4L}}} \right)\ln \left( {\frac{{2L}}{{2H + R}}} \right)}}} \right)$$

*α*_*eff*_ is complicated and the derivation is nontrivial;^30^ it is a function of both *β* and the geometrical properties of the ellipsoid representing the tip. The ellipsoid has an apex radius of curvature *R*, which should correspond to the true experimental tip radius, a characteristic half-length *L*, which does not correspond to the true tip shank length, and a complex parameter *g* which describes the charge distribution on the tip in the presence of incident radiation; specifically, *g* is a measure of the charge concentrated near the tip apex, and therefore represents the proportion of the total induced charge which participates in the near-field interaction. Although complicated, this result has proven to be quite useful, as it connects the measured THz signal to the desired dielectric parameters for a given tip geometry, at least in the case of linear elastic scattering.

**Fig. 5:**
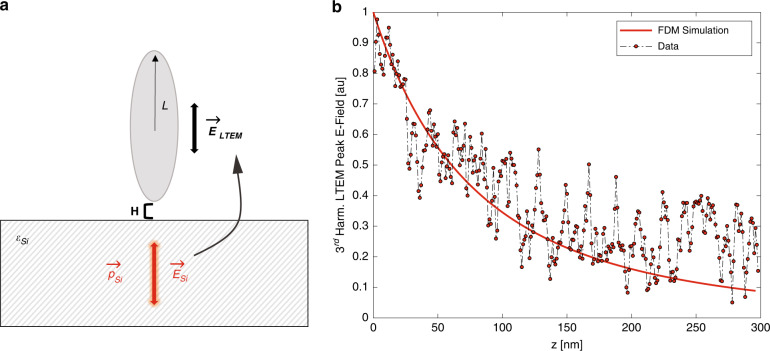
The LTEM Finite Dipole Model. **a** Schematic of the LTEM FDM. The tip (represented as a spheroid of half-length L) is held above a sample with dielectric function *ε*_*Si*_. A subsurface dipole $\vec p_{Si}$ generates a subsurface field $\vec E_{Si}$, which leaks out of the substrate and is felt by the tip as effective field $\vec E_{LTEM}$. **b** Successful parameter tuning of the LTEM FDM (solid line) to a 4th harmonic NIR-induced THz emission approach curve on p-InAs (circular symbols)

To extend this result to the case of THz emission, we change the origin of the polarizing field; in the original FDM, the incident THz pulse is represented as a constant vertical electric field, propagating in free space above the sample. The resulting near-fields at the tip apex are represented by a series of charges and image charges within the spheroid and sample, and an electrostatic method-of-images approach is used to calculate the total radiated field from the system and finally extract the resulting near-field signal strength. In the case of THz emission, the THz field originates not in the air, but from the dielectric material under study. A schematic of this system is shown in Fig. 5a. We therefore impose a vertically oriented subsurface dipole, which generates a constant vertical electric field. We then use the method of images to determine the effective field that interacts with the tip, located on the other side of the dielectric boundary (i.e., in air). We then proceed with the same analysis as used in the finite dipole model^30^, substituting the original incident THz field (*E*_*inc*_), with our calculated final emission field (*E*_*LTEM*_).

Using this approach, one can show that the field experienced by the tip is proportional to the true subsurface field generated below the Si surface (*E*_*Si*_) scaled by the constant $\frac{2}{{\left( {\varepsilon _{Si} + 1} \right)}}$; this is the appropriate image charge scaling constant when the originating field is below the sample surface. Here, *ε*_*Si*_ is the dielectric function of Si^32^. The resulting incident field then, can be written as

3 $$E_{LTEM} = \frac{2}{{\left( {\varepsilon _{Si} + 1} \right)}}E_{Si} = \left( {1 - \beta } \right)E_{Si}$$

This analysis has neglected the fact that the incident (blue) pump pulse is partially reflected at the Si surface. To include this effect, we add the Fresnel transmission coefficient (*t*_*s*_) for s-polarized light, evaluated at the wavelength of the incident pump pulse:^32^

4 $$E_{LTEM} = t_s\left( {1 - \beta } \right)E_{Si}$$

Substituting Eq. (4) into the original expression results in an electrostatic model which relates the dielectric properties of the sample to the emitted THz field coupled to the tip:

5 $$E_{emitted} = t_s\left( {1 - \beta } \right)\left( {1 + \beta } \right)\alpha _{eff}E_{Si}$$

In the measurements described here, we do not know a priori the strength of the field *E*_*Si*_ induced by the optical excitation. However, we may normalize our results to those obtained from a hypothetical sample with very low doping concentration (we choose 10^15 ^cm^−3^). We can then observe the relative change in signal, in each case, to quantify the carrier concentration in the implanted region of the sample.

To implement these models, we set *R* = 40 nm (to represent our real tip radius of 40 nm) and tune parameters *L* and *g* by finding the best fit of the LTEM FDM to the NIR emission approach curve on InAs (Fig. 5b). This approach curve, which relates the THz emission strength in p-InAs to the tip-sample distance, is a comprehensive indicator of the broadband near-field strength and confinement^10^. Therefore, tuning the model to best fit this approach curve will provide a spheroid geometry for the FDM which very accurately recreates our experimental conditions. We find best-fit parameters of *L* = 1.2 µm, and *g* = 0.75e^0.06*i*^, which agree well with values used previously for similar experiments^30^.

### Extracting Si carrier concentration

**Fig. 6:**
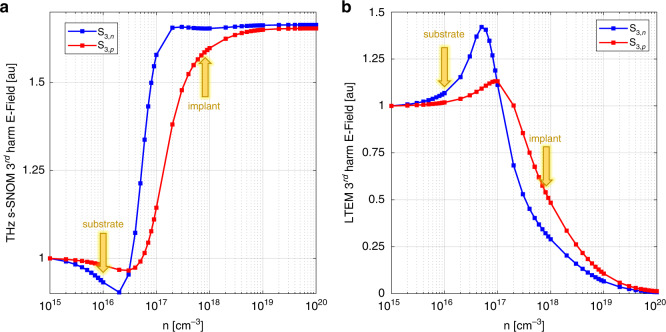
FDM and LTEM FDM predictions of THz near-field signal as a function of Si doping concentration. **a** FDM simulation of the 3rd harmonic THz s-SNOM signal at 0.84 THz vs. local Si carrier concentration, for increasing n doping (blue) and increasing p doping (red). **b** FDM simulation of 3rd harmonic LTEM signal at 0.84 THz from Si, as *n* doping increases (blue) and *p* doping increases (red). Specified substrate doping is marked with a yellow arrow as “substrate”, and each model’s predicted implant doping is marked by a yellow arrow labeled “implant”. Both models predict an approximate implant doping of 8 × 10^17^ cm^−3^

With these parameters fixed, and using the Drude model^33,34^ to calculate the relationship between *n*/*p* doping and *ε*_*Si*_, we use both versions of the FDM discussed above to relate the strength of the scattered field to the local doping density in the sample. The results are shown in Fig. 6. We note that, although the detected pulses are broadband, it is a reasonable approximation to only simulate the scattered or emitted field component at 0.84 THz; this is the strongest frequency component in our detected spectra, and it is much less computationally expensive to do a single-frequency simulation. However, these calculations can easily be expanded to include multiple frequencies in any future experiments where a rigorous spectroscopic approach is required.

As expected, the conventional FDM predicts an increase in THz signal with doping (except for a small decrease between 10^16^ and 10^17 ^cm^−3^ due to plasmonic effects). It is worth noting that the precise quantitative relationship between the doping concentration and the THz s-SNOM signal is a result of tip geometry, the inherent optoelectronic properties of the material under study, and various other experimental conditions. However, our model’s prediction that highly doped Si produces an ~70% greater THz s-SNOM signal than lightly doped Si is consistent with similar calculations in prior works^17,21^.

In contrast, the modified FDM predicts that higher doping results in lower LTEM signal, due to both decreased pump absorption and decreased coupling of radiation from a sub-surface dipole out into free space. To compare our experimental results to these predictions, we first locate the points on the blue curves (for *n* doping) corresponding to the known doping concentration of the native Si substrate (10^16 ^cm^−3^). We then scale these points by the experimentally determined contrast ratios between the implanted and native regions of the wafer (1.7 times increase in THz elastic scattering, 1.9 times decrease in THz emission strength). The two resulting points on the red curves (for *p* doping) both correspond to the carrier concentration of 8 × 10^17^ cm^−3^ (yellow arrows in Fig. 6). This value matches well with the known *p* doping concentration for the implanted region of the sample studied here. This excellent agreement validates the modified FDM and demonstrates that quantitative information can be obtained through blue-light-induced LTEM in wide bandgap semiconductors.

## Discussion

In summary, we present the first example of s-SNOM using a blue light incident wave in the form of blue-pulse-induced terahertz emission. We demonstrate that, because the tip acts as a nano-probe which outcouples only local terahertz emission from a larger macroscopic dipole, we may bypass the alignment challenges typically associated with coupling short-wavelength radiation to the AFM tip; therefore, near-field LTEM is an excellent choice for nanoscale studies of blue-light-induced nonlinear optical phenomena in semiconductors. We compare the emitted broadband THz pulses from lightly doped InAs when pumped with NIR vs blue light pulses. The spectral narrowing in the latter case reveals the differences in carrier mobility in different regions of the conduction band—a property which has not previously been observed with near-field LTEM. Then, by pumping with blue light, we present the first near-field THz emission from bulk Si. We show that the strength of the emission is as sensitive to changes in carrier concentration as a traditional THz s-SNOM measurement. We also present an extension of the well-established finite dipole model for THz s-SNOM in order to predict the relationship between the blue light-induced THz emission from Si and the local carrier concentration. We show that both the traditional and modified FDMs match well to our experimental results. These results suggest that our technique greatly expands the possibilities for LTEM spectroscopy and imaging, providing an avenue to directly observe charge carrier properties in materials which have not so far been amenable to THz s-SNOM techniques.

## Materials and methods

### s-SNOM measurements

Our s-SNOM measurements use a commercial AFM (Neaspec GmbH) operated in tapping mode. We have chosen PtIr-coated conical probe tips (Rocky Mountain Nanotechnology) which are fabricated with a ~40 nm tip radius and 80 μm shank length, though different geometrical parameters may be chosen to tune the imaging resolution or antenna-resonant THz coupling^35,36^.

Ultrafast (~100 fs) near-infrared (NIR) pulses with 820 nm center wavelength are generated by a Ti:Sapphire oscillator (Mai Tai, Spectra Physics), with 80 MHz repetition rate and 2.8 W average power. These pulses are split such that one branch goes directly to the sample for NIR photoexcitation, one enters an optical parametric oscillator (Inspire Auto 100, Spectra Physics) and is frequency doubled to produce ultrashort pulses with 410 nm center wavelength one is incident on a GaAs photoconductive antenna (Tera-SED, Laser Quantum) which produces broadband (0.2–2.2 THz) terahertz pulses for THz s-SNOM measurements, and one is used for electro-optic detection of the time-domain THz waveform using a 1 mm thick ZnTe crystal. Only a very small portion of the Ti:Sapphire output power is required to photoexcite the materials under study and generate substantial THz emission; both NIR and blue pump beams are attenuated down to an average power of ~10 mW. The NIR pump, blue light pump, and PCA-generated THz beams are focused with off-axis parabolic mirrors inside the AFM onto the sample surface and are elastically scattered from the tip-sample system. To ensure proper THz pulse detection, excess NIR or blue radiation which is scattered from the tip towards the EO crystal is blocked with a thin Teflon slab. The AFM tip taps vertically above the sample surface with amplitude 200 nm and frequency ~23 kHz. A lock-in amplifier is referenced to a harmonic of the tapping frequency in order to guarantee sufficient background suppression while maintaining a high enough signal-to-noise ratio^37,38^; the 2nd, 3rd, and 4th harmonic demodulation are normally appropriate and are used for the data presented in this work. By fixing the sample position, we may measure time-domain THz waveforms, or, by fixing the time delay, we may detect the peak scattered/emitted signal and raster-scan the sample to generate THz near-field reflection or emission images. All data are collected in ambient conditions.
